# Clinical, Bacteriologic, and Geographic Stratification of Melioidosis Emerges from the Sri Lankan National Surveillance Program

**DOI:** 10.4269/ajtmh.17-0441

**Published:** 2018-01-08

**Authors:** Harindra D. Sathkumara, Adam J. Merritt, Enoka M. Corea, Shivankari Krishnananthasivam, Mohan Natesan, Timothy J. J. Inglis, Aruna Dharshan De Silva

**Affiliations:** 1 Genetech Research Institute, Colombo, Sri Lanka;; 2 PathWest Laboratory Medicine, QE2 Medical Centre, Nedlands, Western Australia, Australia;; 3 Faculty of Health Sciences and Medicine, Marshall Centre, School of Biomedical Sciences, University of Western Australia, Perth, Western Australia, Australia;; 4 Department of Microbiology, University of Colombo, Colombo, Sri Lanka;; 5 Division of Molecular and Translational Sciences, United States Army Medical Research Institute of Infectious Diseases, Frederick, Maryland;; 6 Faculty of Health Sciences and Medicine, School of Medicine, University of Western Australia, Perth, Western Australia, Australia;; 7 Department of Paraclinical Sciences, Faculty of Medicine, Kotelawala Defense University, Ratmalana, Sri Lanka;; 8 Division of Vaccine Discovery, La Jolla Institute of Allergy and Immunology, La Jolla, California

## Abstract

Melioidosis, a potentially fatal tropical infection, is said to be underdiagnosed in low-income countries. An increase in melioidosis cases in Sri Lanka allowed us to analyze the relationship among clinical outcome, bacteriology, epidemiology, and geography in the first 108 laboratory-confirmed cases of melioidosis from a nationwide surveillance program. The additional 76 cases of laboratory-confirmed melioidosis confirmed further associations between *Burkholderia pseudomallei* multilocus sequence typing (MLST) and infection phenotype; ST1137/unifocal bacteremic infection (χ^2^ = 3.86, *P* < 0.05), ST1136/multifocal infection without bacteremia (χ^2^ = 15.8, *P* < 0.001), and ST1132/unifocal nonbacteremic infection (χ^2^ = 6.34, *P* = 0.02). ST1137 infections were predominantly seen in the Western Province, whereas ST1132, 1135, and 1136 infections predominated in the Northwestern Province. Early participating centers in the surveillance program had a lower melioidosis-associated mortality than later participants (χ^2^ = 3.99, *P* < 0.05). The based upon related sequence types (eBURST) algorithm, a MLST clustering method that infers founding genotypes and patterns of descent for related isolates and clonal complexes in an unrooted tree, showed uneven distribution of sequence types (STs). There was spatial clustering of the commonest STs (ST1132, 1136, and 1137) in the Western, Northwestern, and Central provinces. The recent increase in melioidosis in Sri Lanka uncovered by laboratory-enhanced surveillance is likely to be the result of a combination of improved laboratory detection, increased clinician awareness, recruitment of clinical centers, and small outbreaks. Further development of the surveillance program into a national genotyping-supported melioidosis registry will improve melioidosis diagnosis, treatment, and prevention where underdiagnosis and mortality rates remain high.

## INTRODUCTION

Melioidosis is a potentially fatal bacterial infection resulting from contaminated soil or water exposure in many parts of the tropics.^1^ In recent years, sporadic cases and occasional case clusters of melioidosis have been recognized in many places outside the main endemic region in Southeast Asia and northern Australia.^2–4^ These cases have long been thought to be a fraction of the actual case burden, and most likely reflect underdiagnosis because of the need for a combination of physician awareness, specific clinical laboratory expertise, and infrastructure.^5^ Key difficulties that hampers the early recognition of melioidosis are the broad range of clinical presentations, spanning subclinical seroconversion with delayed onset bacteremia, single or multifocal soft tissue infections, pyogenic infections of specific organs to pneumonia, and septicemia.^6,7^ Melioidosis is, therefore, a cluster of clinical presentations caused by a common pathogen, the select agent *Burkholderia pseudomallei*. Several disease classification schemes have been reported for melioidosis, each with different emphases and priorities.^8–10^ In our first report on a smaller series of culture-confirmed cases from Sri Lanka, we stratified cases on clinicopathologic grounds by whether focal infection was present in one or more organ systems and whether bacteremia was detected.^10^ Our classification schema reduced the wide range of clinical presentations to five generic categories and revealed an association between disease category and *B. pseudomallei* genotype in the unifocal bacteremic group. The recent nationwide increase in culture-confirmed melioidosis cases, coupled with prospective *B. pseudomallei* genotyping, presented an opportunity to investigate the phenotypic, genotypic, and geographic stratification of melioidosis in more detail. In the present study, we sought to develop a phylogeographic appreciation of disease emergence, understand its progression to different clinical outcomes, and measure the extent of disease class/genotype correlation.

## METHODS

### Case notification procedure.

Clinically suspected melioidosis cases were identified through a national clinical microbiology network representing the major government and teaching hospitals in Sri Lanka. Suspected *B. pseudomallei* isolates were prospectively collected from cases of septicemia, pneumonia, or deep abscesses and reported to the Department of Microbiology, Faculty of Medicine, University of Colombo for culture confirmation between February 2014 and December 2015. Clinical and laboratory measurements were recorded using standardized data collection forms.

### Research ethics.

The study was approved by the Institutional Review Board/Ethics Review Committee of the Faculty of Medicine, University of Colombo, Sri Lanka (EC-13-188), and by the Office of Human Research of the United States Army Medical Research and Material Command. Enrolment of study participants whose cultures were positive for *B. pseudomallei* was conditional on appropriate written informed consent administered by a research assistant where applicable.

### Bacterial isolation and identification.

Primary isolation relied on conventional culture techniques for blood and other sterile fluids, sputum, pus, and other specimens from patients with focal pyogenic infection. Bacterial isolates that were oxidase-positive, gentamicin-resistant, and Gram-negative bacilli were forwarded to the reference laboratory in Colombo, where they were subcultured to establish pure growth and maintained at −70°C in 15% brain heart infusion glycerol for subsequent definitive tests. Bacteria were resuscitated by subculture onto 5% blood agar and incubated for 24 hours at 37°C to give single-colony growth. A single blood agar plate was used for each isolate, and stock cultures were spread to produce single-colony growth in the third or fourth quadrant. A single colony was then subcultured and a colony of bacteria was resuspended followed by heat inactivation as described.^11^ The identity of *B. pseudomallei* isolates was confirmed by real-time polymerase chain reaction (PCR) assay, amplifying lpxO gene using the primers and conditions described.^11^ PCR assays were performed in 25 µL reactions and contained 12.5 µL of 2× SYBR Green quantitative PCR (qPCR) master mix (Qiagen, Hilden, Germany), 1 µL of 10 µM forward primer, 1 µL of 10 µM reverse primer, 9.5 µL of molecular biology grade water, and 1 µL of template DNA. In addition, mutually exclusive Yersinia-like fimbrial (YLF) and *B. thailandensis-*like flagellum and chemotaxis (BTFC) gene clusters of *B. pseudomallei* were detected using a multiplex SYBR green real-time PCR assay as previously described.^12^ The assay was conducted as outlined earlier with 1 µL of 10 µM forward and reverse primers for both YLF and BTFC. High-resolution melt curves were analyzed to ascertain product specificity. The reactions were performed on a Rotor-Gene Q (Qiagen) real-time PCR system at Genetech Research Institute.

### Bacterial genotype determination.

The molecular diversity of strains was assessed using the *B. pseudomallei* multilocus sequence typing (MLST) schema. Six of seven housekeeping genes (ace, gltB, gmhD, lipA, narK, and ndh) were amplified using the original primers described previously.^13^ PCR assays were performed in 50 µL reactions with initial denaturation at 95°C for 5 minutes, followed by 35 cycles of 95°C for 30 seconds, 62°C for 30 seconds, and 72°C for 30 seconds. The samples were then maintained at 72°C for a further 10 min and kept at 4°C until the tubes were removed. The other housekeeping gene fragment, lepA was amplified using the primers and conditions described by McCombie et al.^14^ PCR products were then subjected to electrophoresis using a 2% agarose gel to confirm the correct fragment size and purity. Reactions with nonspecific products were discarded and repeated. The purified PCR products were subsequently subjected to downstream automated Sanger sequencing. Bidirectional sequencing reactions were carried out and resolved with an Applied Biosystems 3730xl capillary sequencer (Macrogen, Seoul, South Korea).

### Bioinformatic analysis.

Sequencing chromatograms were interpreted using GeneiousR9 (http://www.geneious.com).^15^ Allele sequences were compared against the reference sequences available in the *B. pseudomallei* MLST database (http://pubmlst.org/bpseudomallei/). All new and existing alleles and sequence types (STs) along with isolate data and sequence traces were submitted to the *B. pseudomallei* MLST database (http://pubmlst.org/bpseudomallei/). The relatedness of MLST profiles of Sri Lankan *B. pseudomallei* strains in the MLST database (as of April 2016) was analyzed using the global optimal based upon related sequence types (eBURST; goeBURST) with single-locus variants (SLVs) selected,^16^ implemented in the PHYLOViZ program.^17^ goeBURST is a refinement of the original eBURST algorithm by Feil et al.^18^ where links that break the rules of the eBURST algorithm are prevented.

For maximum likelihood inference, model selection was performed on the sequences using jmodelTest v 2·1·9 (https://github.com/ddarriba/jmodeltest2).^19,20^ The settings used were as follows: substitution models = 11, +F, +I, and +G for a total of 88 substitution schemes. Bayesian information criteria were used to rank the assessed schemes.^21,22^ The base tree was ML optimized and nearest neighbor interchange was used for the base tree search. MEGA v7.0.18 (http://www.megasoftware.net/) was then used to infer a phylogenetic tree.^22^ The maximum likelihood method was used with the recommended Tamura–Nei model using a discrete gamma parameter (cat = 4, G = 0.0500) and a proportion of invariable sites allowed.^23^ The global *B. pseudomallei* MLST dataset was re-downloaded and manually curated as previously described using the United Nations geoscheme^10^ [last accessed on March 15, 2016]. DnaSP v5·10 (http://www.ub.edu/dnasp/) was again used to assess diversity, divergence, and differentiation.^24^

### Statistical analysis.

Bacteriologic, epidemiologic, and geographic data were collated in a single spreadsheet (TJJI, AJM; Excel, Microsoft) maintained by one of the investigators (EMC). Data were anonymized and transferred for exploratory data analysis to develop hypotheses in accordance with the previously published emerging infectious disease investigation criteria^25^ using data mining software (Orange V2.7; University of Ljubljana, Ljubljana, Slovenia). The two principal data mining strategies used were cluster analysis for numeric variables or numeric/categorical combinations, and decision tree building for categorical analysis. Subsequent statistical analysis was conducted using Fisher’s exact test (Prism, 6·0; GraphPad, San Diego, CA) and an odds ratio (OR) calculator (MedCalc; https://www.medcalc.org/calc/odds_ratio.php).

### Spatial analysis.

Using a map of Sri Lankan provincial boundaries as a base layer (Wikipedia Commons) and Google Map as a location-finding overlay, the culture-positive cases were plotted by place of residence at the time of diagnosis. Geographic information system analysis of genotype data was performed using GenGIS v2·5·0 (http://kiwi.cs.dal.ca/GenGIS/Download) and R v3·3·2 (https://www.r-project.org/).^26,27^ The maximum likelihood tree was projected onto an administrative boundary vector map layer of Sri Lanka (GADM: http://biogeo.ucdavis.edu/data/gadm2.8/shp/LKA_adm_shp.zip). The GIS coordinates of each patient’s home location were plotted on a map of the island and color coded in accordance with the corresponding *B. pseudomallei* goeBURST cluster. The angle of the geographic layout line was determined by the multi-tree optimal-crossing test. The significance of all the possible angles was determined by permutation tests with 10,000 replicates, using a critical *P* value of 0.001. The fit of leaf nodes to geographic points was determined by using the Monte Carlo permutation test (1,000 replicates) at the selected angle. Canonical correlation analysis was performed to identify correlations between latitude/longitude of isolation site and infection type or eBurst cluster. Correlations were then investigated by using the Mantel test.

## RESULTS

**Figure 1.**
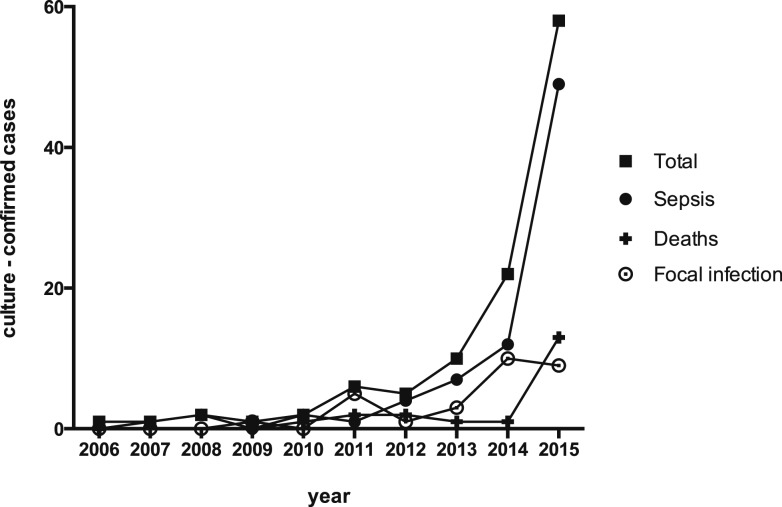
Culture-confirmed melioidosis in Sri Lanka by year from 2006, including total cases, fatalities, sepsis (S0, SS, and SM), and focal infections (FS and FM).

The first culture-confirmed case of melioidosis in the current Sri Lankan series occurred in 2006. Since then, there has been an exponential growth in total and fatal culture-confirmed cases (Figure 1). Building on our initial *B. pseudomallei* collection, isolates from 76 culture-confirmed cases of melioidosis were identified between February 2014 and December 2015 (Supplemental Table 2). The MLST analysis revealed three new allele sequences: one gmhD (allele 124) and two ndh (alleles 20 and 58). Our collection of new strains (76 isolates) resolved to a total of 36 STs. Twenty-three novel STs were shared between 31 isolates and demonstrated greater diversity. Thirteen STs were already present in the *B. pseudomallei* MLST database, of which eight STs had been seen before in Sri Lanka.^10^ ST1137 remained the most common ST in Sri Lanka, representing 13 of 76 isolates.^10^ There were five shared STs (10 isolates), of which three were exclusively seen in the Southeast Asian region (ST308, ST655, and ST912). Five isolates belonged to ST594, which has been seen among clinical and environmental isolates in both Australia and Thailand. One of our isolates (BPs102) belonged to ST132, a dominant Australian ST seen among a large number of clinical, animal, and environmental isolates (*N* = 139 as of April 2016). However, this was found to carry the YLF gene cluster found predominantly among isolates of Southeast Asian origin.

The unifocal bacteremia group was at an increased risk of sepsis and associated with a reduced risk of lower limb infection, but not of death (Table 1). The group that had multifocal infection with bacteremia did not have a significant association with reported sepsis or death, but there was an association with increased risk of lower limb infection. OR analysis of the most commonly encountered clinical presentations showed that an absence of any identifiable abscess was associated with increased risk of sepsis, whereas joint infections and liver abscesses were associated with reduced risk of sepsis. The absence of an abscess and lower respiratory tract infection were both associated with increased odds of death. The odds of lower respiratory infection were higher in patients with no abscesses and reduced if a single abscess was present. Blood culture-negative infections also increased, but remained in a minority.

**Table 1:** Clinicopathologic categories, clinical presentations, and correlated outcomes of melioidosis

		Sepsis				Death				LL* infection				LRTI		
Infection†	*n*	OR‡	95% CI§	*P*	*n*	OR	95% CI	*P*	*n*	OR	95% CI	*P*	*n*	OR	95% CI	*P*
FS	15	0.012	0.0007–0.21	0.002	2	0.50	0.10–2.4	NS	5	1.2	0.38–3.9	NS	2	0.27	0.06–1.2	NS
FM	14	0.013	0.0008–0.21	0.003	2	0.54	0.11–2.6	NS	7	2.8	0.88–8.6	NS	4	0.78	0.22–2.7	NS
S0	5	7.0	0.38–130	NS	2	2.4	0.39–16	NS	1	0.58	0.06–5.4	NS	0	0.17	0.009–3.1	NS
SS	46	11	3.7–30.5	< 0.001	12	1.5	0.59–3.7	NS	0	0.01	0.001–0.17	0.002	18	1.6	0.70–3.5	NS
SM	28	2.7	0.99–7.4	NS	5	0.94	0.33–2.7	NS	19	5.1	1.7–15	0.003	12	1.8	0.72–4.2	NS
Sepsis	68	–	–	–	17	1.6	0.59–4.2	NS	18	0.60	0.26–1.4	NS	25	1.5	0.65–3.6	NS
SSh	2	–	–	–	0	0.75	0.04–16	NS	1	2.4	0.15–40	NS	1	1.9	0.12–32	NS
LRTI	36	1.5	0.65–3.6	NS	36	3.9	1.5–10	0.004	8	0.43	0.16–1.1	NS	36	–	–	–
Joint	19	0.77	0.28–2.1	NS	2	0.36	0.08–1.7	NS	17	39	8.1–185	< 0.001	5	0.67	0.22–2.0	NS
CNSI	6	3.0	0.33–26	NS	3	3.9	0.72–20	NS	2	1.1	0.20–6.6	NS	1	0.38	0.04–3.4	NS
UGI	6	8.4	0.46–154	NS	3	3.9	0.72–20	NS	0	2.1	0.63–7.0	NS	1	0.38	0.04–3.4	NS
Abs, Liv	18	0.16	0.05–0.51	0.002	4	1.0	0.30–3.4	NS	3	0.40	0.11–1.5	NS	4	0.52	0.16–1.7	NS
Abs, Ps	6	0.27	0.05–1.6	NS	1	0.69	0.08–6.2	NS	–	–	–	–	1	0.38	0.04–3.4	NS
Abs, > 1	9	0.44	0.11–1.1	NS	3	1.9	0.43–8.1	NS	4	1.9	0.48–7.7	NS	4	1.7	0.42–6.6	NS
Abs, 1	43	0.51	0.23–1.1	NS	3	0.16	0.043–0.57	0.005	16	1.7	0.73–3.8	NS	5	0.14	0.05–0.41	< 0.001
Abs, 0	56	2.5	1.1–5.6	0.024	18	3.6	1.3–10	0.013	13	0.048	0.21–1.1	NS	27	4.4	1.83–11	0.001

NS = non significant.

* LL = lower limb disease.

† Abs, Liv = liver abscess; Abs, Ps = psoas abscess; Abs, > 1 = multiple abscesses; Abs, 1 = single abscess; Abs, 0 = no abscess identified; CNSI = central nervous system infection; FM = multifocal and not bacteremic; FS = unifocal and not bacteremic; LRTI = lower respiratory tract infection; S0 = bacteremic with no identifiable organ system focus; SM = bacteremic with multiple organ system foci; SS = bacteremic with single organ system focus; Sepsis as reported by the requesting physician; SSh = septic shock; UGI = urogenital infection.

‡ OR = odds ratio.

§ CI = confidence interval.

A breakdown of the five classes of infection by their commoner clinical presentations revealed several significant associations between multifocal infection without bacteremia and liver abscess (χ^2^ = 7.73, *P* = 0.02), unifocal infection with bacteremia and urogenital tract infection (χ^2^ = 6.14, *P* < 0.01), and multifocal infection with bacteremia and joint infection (χ^2^ = 14, *P* < 0.001), but not between unifocal bacteremic infection and lower respiratory tract infection (χ^2^ = 0.70, not significant). A breakdown of disease class by commonest occupational groups, comorbid disease state, and reported environmental exposure revealed no significant association with disease phenotype (Table 2).

**Table 2:** Features of the major clinicopathologic types of melioidosis

Class*	Total	Died	Major clinical presentations								Comorbidities and exposure risks									*B. pseudomallei* genotypes						
			Sepsis	SS†	LRTI	CNSI	UGI	Abs, Liv	Abs, Ps	Joint	Diabetic	Alcohol	CKD	Rice	Garden	Floods	Farmer	Housewife	Driver	1132	1135	1136	1137	1140	1434	Other ST
FS	15	2	0	0	2	0	0	4	1	2	11 (2)‡	1	1	0	1	0	1	3 (1)	1	4 (1)	3 (1)	0	1	0	0	7
FM	14	2	0	0	4	0	0	6	2	4	9 (2)	2	1	3 (1)	3	0	3 (2)	1	2	0	1	5 (1)	2	1	0	5 (1)
S0	5	2	5	1	0	0	0	0	0	0	3 (1)	3 (2)	0	1	3 (1)	1	0	1	1	0	1 (1)	0	1 (1)	0	0	3
SS	46	12	42	1	18	3	6	5	1	2	26 (6)	4 (1)	5 (1)	16 (3)	8 (1)	7 (1)	10 (4)	10 (3)	4	2	3 (1)	1	12 (4)	2	3 (1)	23 (6)
SM	28	6	22	0	12	3	0	2	2	11	18 (3)	3 (1)	1	9 (1)	3 (2)	3 (2)	7	3	5	2	1	2 (1)	2	2	3	16 (5)
Total	108	24	69	2	36	6	6	17	6	19	67 (14)	10 (4)	8 (1)	28 (5)	18 (4)	11 (3)	21 (6)	18 (4)	13	8 (1)	9 (3)	8 (2)	18 (5)	5	6 (1)	54 (12)

* Clinicopathologic classification: FM = multifocal and not bacteremic; FS = unifocal and not bacteremic; S0 = bacteremic with no identifiable organ system focus; SS = bacteremic with single organ system focus; SM = bacteremic with multiple organ system foci.

† Septic shock.

‡ Numbers in brackets indicate melioidosis-associated deaths.

*B. pseudomallei* isolates from the expanded collection of culture-confirmed cases of melioidosis belonged to a total of 46 sequence types, the commonest of which were STs 1137, 1135, 1132, 1136, 1434, and 1140. These six genotypes accounted for 54 (50%) isolates and 12 (50%) deaths. The commonest ST 1137 retained its previously reported association with unifocal bacteremic infection (χ^2^ = 3.86, *P* < 0.05).^10^ Two other significant ST–disease phenotype associations were apparent from this series: ST 1136 (χ^2^ = 15.8, *P* < 0.001) and multifocal infection without bacteremia, and ST 1132 and unifocal non-bacteremic infection (χ^2^ = 6.34, *P* = 0.02).

Nucleotide diversity (π) for our expanded set of Sri Lankan isolates was 0.00136 compared with 0.00253 for the rest of world population. Measures of divergence and differentiation between the Sri Lankan and the rest of world *B. pseudomallei* population were *D*_XY_ = 0.00245 and *F*_ST_ = 0.201 (previously *D*_XY_ = 0.00252 and *F*_ST_ = 0.246). Southern Asian isolates remained the least divergent from Sri Lankan ones, and the divergence and differentiation measures reduced to 0.00163 and 0.06, respectively (previously 0.00209 and 0.273).^10^

**Figure 2.**
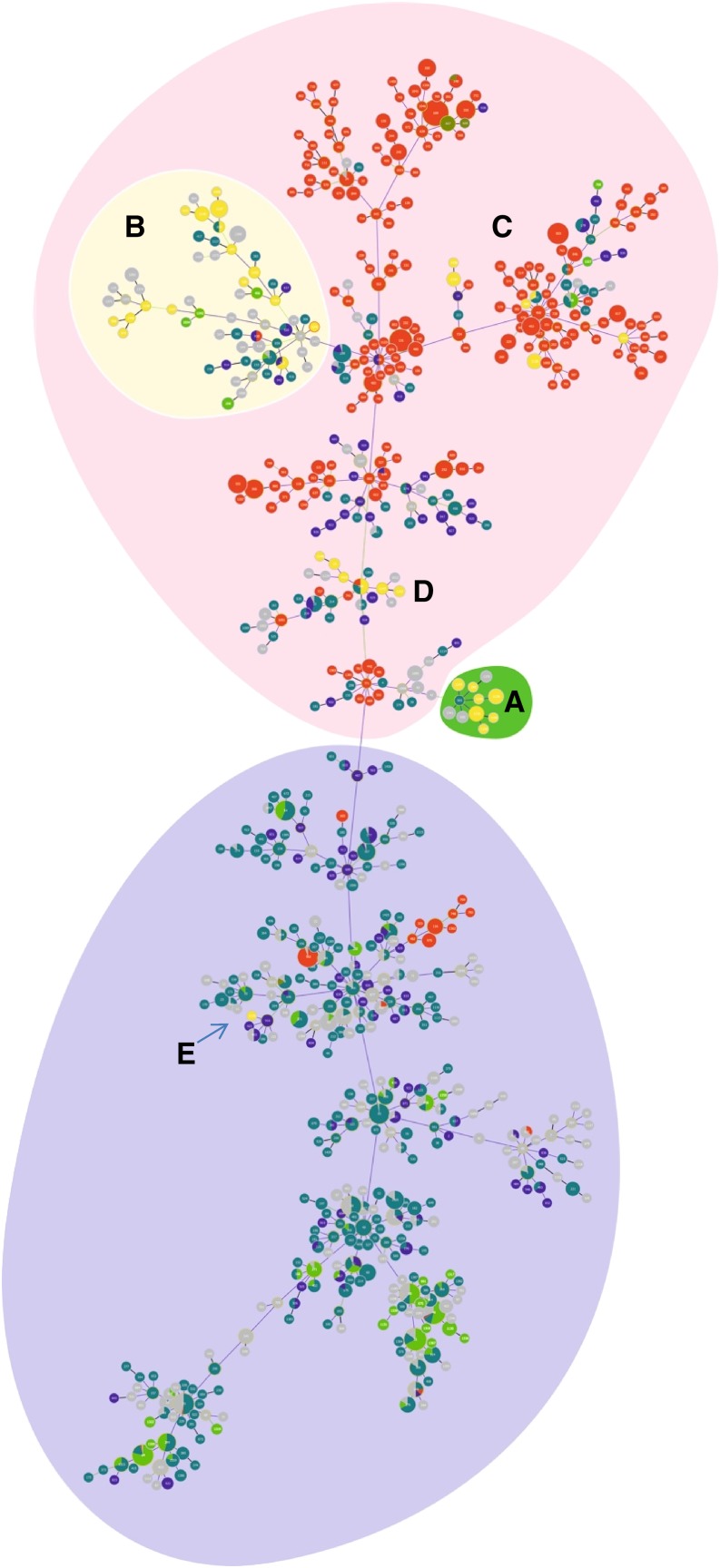
Global optimal based upon related sequence types (goeBURST) population snapshot of *Burkholderia pseudomallei*. Grouping was performed at the single-locus variant level. Each dot represents a distinct sequence types (ST). Sri Lankan STs are colored in yellow, whereas STs of Australian origin are in red. Different green and blue dots represent STs found in Malaysia, Thailand, and Cambodia. Oceania and Southeast Asian ST dominant regions are shaded in red and purple, respectively. Groups A (shaded in green) and B (shaded in faded yellow) represent the largely intact original Sri Lankan cluster and new main Sri Lankan branch, respectively. Small group of Sri Lankan STs within Oceania falls into Group C, whereas Group D is composed of Sri Lankan STs within the main branch of the tree. Group E represents the Sri Lankan ST in the Southeast Asia dominant region of the complex. This figure appears in color at www.ajtmh.org.

The phylogenetic relationship between 95 isolates and curated *B. pseudomallei* sequence types from Southeast Asia and Australasia is shown in the new goeBURST analysis (Figure 2). The addition of new isolates did not greatly alter the structure of the optimized tree, and the original Sri Lankan cluster located at the midpoint between the Oceania and “rest of world” branches remained largely intact with the addition of one ST (194) and the loss of two (1137 and 1140)—Group A. However, all of the new strains were placed in the Australian region of the diagram, and most (15/23)of these STs were placed in a branch dominated by isolates sourced from many Southeast Asian countries, including Thailand, Malaysia, China, Cambodia, and India—Group B. The remaining STs were unevenly dispersed through the tree, with a small group (STs 202, 590, 1135, 1314, 1434, and 1436—Group C) placed in a branch with other Australian STs. A small set of STs was placed in the main branch of the tree containing STs 13, 594, 1143, 1148, 1152, and 1413—Group D. The single ST (1133) that previously fell on the Southeast Asia dominant side of the tree remains the only ST in that part of the tree—Group E. An additional 13 STs could not be placed in the main clonal group using the SLV setting and are not shown—Group F.

**Figure 3.**
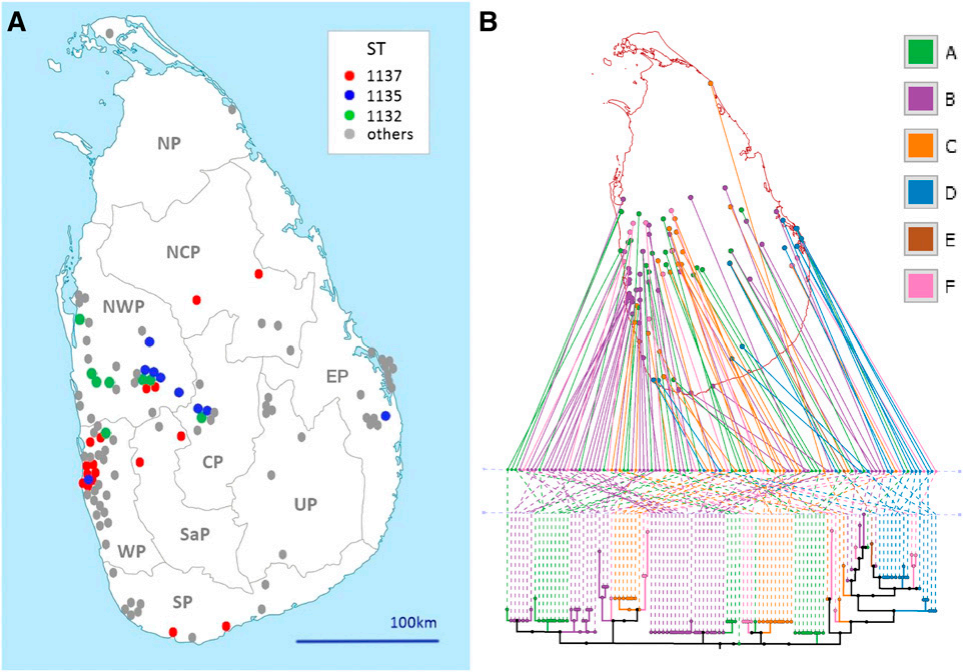
(**A**) Map of Sri Lanka depicting the location of culture-confirmed cases of melioidosis between 2006 and 2015, with the three commonest *Burkholderia pseudomallei* genotypes (sequence types) color-coded to display the extent of spatial aggregation. (**B**) GenGIS projection of a maximum likelihood tree (TN93 model) of 108 Sri Lankan *B. pseudomallei* isolates onto the Sri Lankan administrative area vector map. Layout line angle = 90.4°, crossing count = 1,565. A (green) = previously described Sri Lankan global optimal based upon related sequence types (goeBURST) group,^10^ B (purple) = Southeast Asian goeBURST group, C (orange) = Australian goeBURST group, D (blue) = Sri Lankan new main branch group, E (brown) = ST1133–sole “rest of world” group isolate, and F (pink) = not in the main clonal goeBURST group. This figure appears in color at www.ajtmh.org.

The geographic distribution of patients’ bacterial genotypes was concentrated around the major settlements in the Western Province, stretching into the Northwestern Province, with smaller clusters of patients with culture-confirmed infections in the Central, Eastern, Southern and Uva provinces (Figure 3A, Table 3). There was some geographic clustering of three of the commonest *B. pseudomallei* STs, with ST 1137 concentrated in the vicinity of Colombo. There was a significant difference between the range of common *B. pseudomallei* STs detected in patients from the Western Province (including ST 1137) and those detected in the adjacent Northwestern Province (STs 1132, 1135, and 1136), the provinces with the greatest confirmed melioidosis burden (NWP v WP χ^2^ = 17.75, *P* < 0.001; Table 4).

**Table 3:** Distribution of cases by province

Class	Total	CP*	EP	NCP	NP	NWP	SP	SaP	UP	WP
FS	15 (2)	1 (0)	1 (1)	1 (0)	0	8 (1)	1 (0)	0	0	3 (0)
FM	14 (2)	0	0	1 (0)	0	4 (0)	0	1 (0)	4 (2)	3 (0)
S0	5 (2)	0	1 (0)	0	0	0	0	0	0	4 (2)
SS	46 (12)	4 (0)	8 (2)	3 (2)	0	9 (3)	4 (2)	2 (1)	3 (1)	12 (2)
SM	28 (6)	1 (0)	5 (2)	0	0	9 (2)	3 (0)	1 (1)	0	8 (1)
Total	108 (24)	6 (0)	15 (5)	5 (2)	0	30 (6)	8 (2)	4 (2)	7 (3)	30 (5)

* Provinces of Sri Lanka: CP = Central Province; EP = Eastern Province; NCP = North Central Province; NWP = Northwestern Province; SP = Southern Province; SaP = Sabaragamuwa Province; UP = Uva Province; WP = Western Province.

**Table 4:** Relative occurrence of *Burkholderia pseudomallei* genotypes (STs) by province

ST	Total	Died	CP	EP	NCP	NP*	NWP	SP*	SaP	UP	WP
1132	8	1	1	0	0	0	6 (1)‡	0	0	0	1
1135	9	3	2	1 (1)†	0	0	5 (1)	0	0	0	1 (1)
1136	8	2	1	0	1	0	3 (1)	1	0	2 (1)	0
1137	18	5	0	0	2 (1)	0	2	2 (2)	2 (1)	0	10 (1)
1140	5	0	0	0	0	0	0	0	0	0	4
1434	6	1	0	0	1 (1)	0	0	2	0	0	3

ST = sequence types.

* Provinces of Sri Lanka: CP = Central Province; EP = Eastern Province; NCP = North Central Province; NWP = Northwestern Province; SP = Southern Province; SaP = Sabaragamuwa Province; UP = Uva Province; WP = Western Province.

† Two infections were from patients resident in both Northern and Southern provinces and have been omitted from this table.

‡ Numbers in brackets indicate melioidosis-associated deaths.

The GenGIS-based phylogeography of culture-confirmed melioidosis is shown in Figure 3B. Linear axis angles with less than 1,953.5 crossing were significant and the global optimal angle was found to be 90.74 (1,565 crossings). The relationship between leaf nodes and geographic location was significantly better than the null hypothesis of random chance (*P* value < 0.001). Isolates from eBURST Groups A and F tended to be from the middle latitudes of the country. Group B isolates were concentrated on the west and south coasts with proportionally fewer isolates inland. Group C was mostly restricted to the west, with the exception of one isolate from the far northeast of the country. Group D was most common on the east coast with a few isolates originating further south. In the goeBURST tree, Group D (STs 13, 594, 1143, 1148, 1152, and 14130) was the only ST group to obtain significant statistical support in both longitude and geographic distance (Mantel test, *P* < 0.001). The BTFC variant of *B. pseudomallei* was overrepresented in the Eastern Province (EP) and to a lesser extent in the Northwestern Province (EP v rest of Sri Lanka; χ^2^ = 11.2, *P* = 0.001). A maximum likelihood tree generated from study isolate MLST sequences is shown in Supplemental Figure 1.

The distribution of cases and deaths by province is presented in Table 4. The early clinical centers to adopt the melioidosis surveillance program were located in the Western, Northwestern, and Central provinces. There was a significant difference in melioidosis-associated mortality between these and the later entrants to the surveillance program (χ^2^ = 3.99, *P* < 0.05).

## DISCUSSION

The recent expansion in numbers of culture-confirmed cases follows almost a decade of raising clinical awareness and matching it with improved in-country laboratory capability.^10^ The increase in less than a year from a previous total of 37 to almost three times that number is remarkable. We recognize that this may reflect increased reach of the surveillance program as a result of external funding and reference laboratory support. The proportionate increase in fatal cases demonstrates that improved laboratory support does not always result in faster directed antimicrobial therapy, as observed in our earlier report. Case fatality rates decrease only when clinical awareness is raised above a threshold, where clinicians begin to suspect the infection early and start appropriate therapy while investigations are still in progress. In the early stages of sepsis, the diagnosis is entirely laboratory-based and often comes too late to institute direct therapy, especially when relying on positive blood cultures.

Confirmation of an association between clinicopathologic phenotype and MLST genotype in our patient cohort is further evidence for clade-specific virulence, first proposed in an earlier Australian study which highlighted a potential association with neuromelioidosis.^28,29^ In the present study, there were only six cases of neuromelioidosis and these were attributed to different sequence types of *B. pseudomallei*. What are particularly intriguing about the associations observed in this investigation are the inverse correlations between unifocal and multifocal bacteremic infections and lower limb infection (septic arthritis, psoas abscess, and other soft tissue infections). We noted that lower limb infection was associated with an increased OR (9.95) for multifocal bacteremic infection and a reduced OR for unifocal infection (0.02). Moreover, clinically reported sepsis was associated with a reduced OR for lower limb infection. Both joint infections and liver abscesses had a reduced OR for sepsis. The only clinical presentations we found with an increased odds for death as the major outcome were the absence of an identifiable abscess (OR = 3.5) and lower respiratory tract infection (OR = 3.95).The increased risk of death from respiratory melioidosis has been previously reported^7,30^ and appears to have followed a different pattern to lower limb infection, consistent with an alternative route of bacterial exposure. The S0 class may represent either rapidly progressive sepsis without sufficient time to identify focal organ-specific infection, short-lived bacteremia, or an occult focus such as prostatic abscess, which has been observed in other studies.^31^ The two main bacteremic categories (SS and SM) appear to capture two distinct patterns of pathophysiology in which unifocal bacteremic patients do not localize infection as readily as multifocal bacteremic patients do. We hypothesize that the differences in risk of lower limb infection between these groups reflect a propensity to localize infection in the lower limbs after direct inoculation, resulting in a slowing or limited spread beyond tissues at the point of entry. The subcategories were too small to perform multivariate analysis on the types of environmental or occupational exposure or the possible role of underlying comorbidities such as diabetes, which was common in this series.

A further constraint on our analysis of septicemic melioidosis has been the lack of a consensus definition of sepsis over the last decade. The most widely used clinical definition in Sri Lanka during this period relied on the presence of fever and other common signs of sepsis, such as tachycardia. We did not have access to the additional information required to compile SOFA scores and were not able to stratify patients into systemic inflammatory response syndrome and severe sepsis groups, both of which have been removed from the new international consensus definition of sepsis.^32^ The two remaining clinical categories of sepsis and septic shock are more easily applied in resource-limited tropical health-care settings. We propose the use of the truncated SOFA score (qSOFA) described in the Sepsis-3 guidelines to improve the initial clinical recognition of septicemic melioidosis and advocate its incorporation in melioidosis disease notification or registry schemes such as the Pahang melioidosis registry.^33^

The commonest MLST sequence type (ST 1137) detected in the present study was associated with unifocal bacteremic infection, over half of which (56%) formed a tight spatial cluster in Colombo. Two other STs (1132 and 1135) formed looser spatial clusters in the neighboring Northwest Province. There was a significant difference in the overall composition of common STs between these two provinces. The Eastern and Southern provinces, on the other hand, were notable for a wider diversity of their *B. pseudomallei* STs. The practical consequence of the dominance of a smaller range of STs and their associated clinical presentations in the west of Sri Lanka is that early recognition will occur in close proximity to much of the clinical laboratory infrastructure. Clearly, the converse also applies, and appears to convert into a higher risk of death with melioidosis in those provinces in the south, east, and north of the country. The current exponential increase in documented culture-confirmed cases will enable multivariate analysis and mathematic modeling of specific disease phenotypes, clinical presentations, *B. pseudomallei* STs, and their distribution, ecology, dynamics, and underlying causes in the future.

We previously reported a nucleotide diversity (π) for the Sri Lankan isolates of 0.00124 compared with 0.00253 for the rest of the world population. The increase in diversity of the new dataset is expected given the addition of new isolates. In addition, the decreased differentiation and divergence with isolates from Southern Asia are geographically consistent. Furthermore, the goeBURST analysis shows that some of these STs provide links for the STs of Indian isolates, suggesting a possible ancestral link or point of origin of Sri Lankan isolates. However, the recent publication of MLST analysis of *B. pseudomallei* from cases of melioidosis in Southern India, the closest part of the subcontinental mainland to Sri Lanka, showed substantial ST diversity and no overlap with the Sri Lankan STs.^34^ Additional MLST work in both Sri Lanka and Southern India may reveal STs that are present in both locations, but the current data do not support recent migration or sharing of STs.

A number of novel strains, STs 1364, 1435, 293, and 1140 in particular, have provided branch points or linker STs to other STs and assisted the branch rearrangement within the complex.^14^ The new STs are not evenly dispersed; instead, they form groups with varying degrees of relatedness. Isolates from the set groups seem to have some level of geographic correlation, with one group having a significantly restricted range. Interestingly, almost all the new isolates described here cluster within the Australian dominant side of the tree, with none of the new STs being allocated to the rest of the world region of the tree. This result was at odds with expectations and the dominance of the YLF type in Sri Lanka and raises questions about either the validity of the typing and clustering method or the origins and spread of *B. pseudomallei* in this region. Finally, an Australian exclusive ST, ST132, was seen in an isolate from a 59-year-old farmer with a known history of diabetes. ST132 has not been seen previously outside Australia. The isolate was shown to belong to the YLF type. Previous confirmation of homoplasy within the typing scheme suggests that this may also be the most likely explanation for this case.^35^

For the meantime, it is of interest to note that the geographic distribution of culture-confirmed melioidosis corresponds closely to population density with the notable exceptions of the central upland area and the northern third of the country. Reasons for an absence or low prevalence in these areas have been discussed previously.^10^ However, the spatial correlation between laboratory-confirmed melioidosis and population density introduces another possible explanation for the recent increase in cases in 2014–2015, because these locations also correspond to a recent building, renovation, and civil engineering boom. It is, therefore, of interest that established cities with extensive protected sites, such as Anuradhapura, have been the location of few if any cases despite rice farming and large reservoirs. Not only have major building and civil engineering works caused much topsoil disturbance in recent years, but there has also been widespread peri-urban infill along major arterial routes linking towns in the west of the country so that former rice fields have given way to shops, houses, and domestic gardens. ST number order does denote biological significance. Moreover, allocation of an ST number to a new set of alleles detected in a Sri Lankan isolate has mainly been in order of discovery, which is in turn the consequence of a series of ecologic, pathophysiologic, and laboratory events.^4,10^ The revision of the international *B. pseudomallei* MLST database following the inclusion of whole genome sequence-derived MLST data can be expected to cause its own infill disturbance, as a new bioinformatic regimen settles in.

We conclude that the expanded number of melioidosis cases uncovered in Sri Lanka through an externally funded laboratory-enhanced surveillance program cannot be attributed to any single factor. In addition to improved laboratory detection, the increase in cases is likely to be the result of a combination of increased clinician awareness, recruitment of clinical centers, and small outbreaks. Nevertheless, the addition of almost double the previous total number of cases to the database allowed the detection of additional clinicopathologic, genotypic, and geographic stratification. Further refinement of the current national surveillance program by incorporating Sepsis-3 criteria into the data collected for a melioidosis registry, and whole genome-based genotyping, will provide future opportunities to improve diagnosis, treatment, and prevention of this infection in those parts of Sri Lanka where it retains a tenacious hold on the population.

## Supplementary Material

Supplemental Table.
