# Supplementary material for: Clinical, Bacteriologic, and Geographic Stratification of Melioidosis Emerges from the Sri Lankan National Surveillance Program
Source: Am J Trop Med Hyg. 2018 Jan 8;98(2):607–15. doi: 10.4269/ajtmh.17-0441 (PMC5929190; doi:10.4269/ajtmh.17-0441)
Supplement: Supplementary file 1 [file tpmd170441.SD1.pdf]

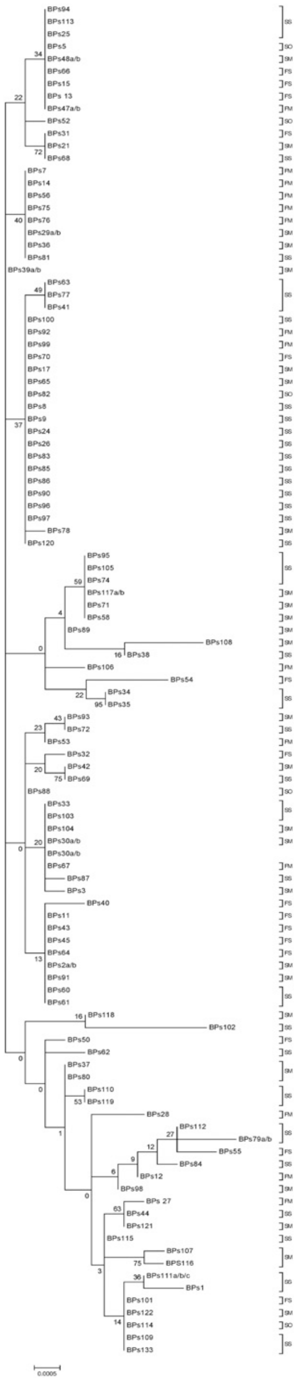

SUPPLEMENTAL FIGURE 1. Maximum likelihood tree of Sri Lankan clinical culture collection. The evolutionary history was inferred using the maximum likelihood method based on the Tamura–Nei model.[23] The tree with the highest log likelihood (−5,128.7110) is shown. The percentage of trees in which the associated taxa clustered together is shown next to the branches. Initial tree(s) for the heuristic search were obtained automatically by applying Neighbor-Join and BioNJ algorithms to a matrix of pairwise distances estimated using the maximum composite likelihood approach, and then selecting the topology with superior log likelihood value. A discrete gamma distribution was used to model evolutionary rate differences among sites (four categories [+*G*, parameter = 0.0500]). The rate variation model allowed for some sites to be evolutionarily invariable ([+*I*], 49.60% sites). The tree is drawn to scale, with branch lengths measured in the number of substitutions per site. The analysis involved 109 nucleotide sequences. All positions containing gaps and missing data were eliminated. There were a total of 3,401 positions in the final dataset. Evolutionary analyses were conducted in MEGA7.[22]

Supplemental Table 1

The largest clinico-pathological group (SS) had a single organ system focus and a positive blood culture. None of these had a significantly raised or lowered odds ratio for the commonly cited melioidosis risk factors. The lack of significant association between these risk factors and the SS clinico-pathological category of infection only indicates a lack of demonstrable stratification within the group.

Infection	Association	χ^2	*P*
Farmer	SS	1.42	NS
Housewife	SS	0.84	NS
Diabetic	SS	0.86	NS
Alcohol	SS	0.02	NS
Rice	SS	1.78	NS
Gardening	SS	0.02	NS
Floods	SS	1.30	NS

NS = non significant; SS = blood culture-positive sepsis with single organ system focus. There is a lack of significant association between main epidemiologic descriptors and the commonest major clinicopathologic class (SS).

Supplemental Table 2

Isolate ID, year of case, loci alleles, and ST for isolates included in the study

ID	Isolate	Year	Loci							ST (MLST)
			ace	gltB	gmhD	lepA	lipA	narK	ndh	
344	BPs42	2014	1	1	6	2	1	1	1	912
353	BPs43	2014	1	12	6	2	1	1	3	1132
366	BPs44	2014	4	4	14	2	1	2	1	13
422	BPs47a	2014	1	2	6	4	1	2	57	1135
684	BPs48a	2014	1	2	6	4	1	2	57	1135
689	BPs50	2014	1	2	3	3	1	2	3	308
693	BPs52	2014	1	12	6	4	1	2	3	194
701	BPs53	2014	1	4	6	2	1	1	3	293
712	BPs54	2014	1	4	13	2	1	1	58	338
989	BPs55	2014	4	12	3	4	1	1	20	474
990	BPs56	2014	1	2	6	2	1	2	57	1136
1024	BPs62	2014	1	2	3	4	1	2	57	590
1030	BPs64	2014	1	12	6	2	1	1	3	1132
1031	BPs79	2015	4	4	3	4	3	1	20	598
1073	BPs82	2015	1	2	6	2	1	8	3	1137
1117	BPs87	2015	4	2	14	2	1	1	3	615
1118	BPs90	2015	1	2	6	2	1	8	3	1137
1141	BPs95	2015	1	2	14	2	5	2	1	1434
1164	BPs104	2015	4	2	6	2	1	1	3	1140
1183	BPs105	2015	1	2	14	2	5	2	1	1434
1236	BPs106	2015	1	4	6	2	5	8	3	733
1322	BPs107	2015	4	4	10	2	1	8	11	867
1333	BPs108a	2015	1	4	14	4	3	42	1	944
1578	BPs109	2015	1	4	3	2	1	2	1	594
1808	BPs110	2015	1	4	3	4	1	2	3	1152
1881	BPs113	2015	1	2	6	4	1	2	57	1135
1929	BPs114	2015	1	4	3	2	1	2	1	594
2116	BPs116	2015	4	4	10	2	1	8	20	1179
2117	BPs117	2015	1	2	14	2	5	2	1	1434
2118	BPs118	2015	1	2	14	4	1	8	1	1314
2119	BPs119	2015	1	4	3	4	1	2	3	1152
2123	BPs120	2015	1	2	6	2	1	8	3	1137
2129	BPs121	2015	4	4	14	2	1	2	1	13
2130	BPs122	2015	1	4	3	2	1	2	1	594
2131	BPs45	2014	1	12	6	2	1	1	3	1132
2132	BPs68	2015	8	2	6	4	1	2	3	1139
2133	BPs75	2015	1	2	6	2	1	2	57	1136
2135	BPs92	2015	1	2	6	2	1	8	3	1137
2136	BPs93	2015	1	4	6	2	1	1	1	1435
2139	BPs103	2015	4	2	6	2	1	1	3	1140
2140	BPs111	2015	1	4	3	2	1	1	1	1364
2141	BPs115	2015	4	4	3	2	1	2	1	1413
2151	BPs133	2015	1	4	3	2	1	2	1	594
2155	BPs112	2015	4	4	3	4	1	1	20	1442
3422	BPs2a/b	2006	1	12	6	2	1	1	3	1132
3423	BPs1	2007	1	4	10	1	1	1	1	1133
3424	BPs3	2008	4	12	6	2	1	1	3	1134
3425	Bps5	2008	1	2	6	4	1	2	57	1135
3426	BPs7	2009	1	2	6	2	1	2	57	1136
3427	BPs8	2010	1	2	6	2	1	8	3	1137
3428	BPs9	2010	1	2	6	2	1	8	3	1137
3429	BPs11	2011	1	12	6	2	1	1	3	1132
3430	BPs12	2011	4	4	3	4	1	1	3	1138
3431	BPs13	2011	1	2	6	4	1	2	57	1135
3432	BPs15	2011	1	2	6	4	1	2	57	1135
3433	BPs17	2011	1	2	6	2	1	8	3	1137
3434	BPs21	2011	8	2	6	4	1	2	3	1139
3435	BPs24	2012	1	2	6	2	1	8	3	1137
3436	BPs25	2012	1	2	6	4	1	2	57	1135
3437	BPs26	2012	1	2	6	2	1	8	3	1137
3438	BPs27	2012	55	4	14	2	1	2	1	1148
3439	BPs28	2013	4	2	3	4	1	2	1	202
3440	BPs29	2013	1	2	6	2	1	2	57	1136
3441	BPs30	2013	4	2	6	2	1	1	3	1140
3442	BPs30b	2014	4	2	6	2	1	1	3	1140
3443	BPs31	2013	8	2	6	4	1	2	3	1139
3444	BPs32	2013	1	2	14	2	1	1	1	1141
3445	BPs33	2013	4	2	6	2	1	1	3	1140

(continued)

Supplemental Table 2
Continued

ID	Isolate	Year	Loci: ace	gltB	gmhD	lepA	lipA	narK	ndh	ST (MLST)
3446	BPs34	2013	1	12	13	2	5	2	58	1142
3447	BPs35	2013	1	12	13	2	5	2	58	1142
3448	BPs36	2013	1	2	6	2	1	2	57	1136
3449	BPs37	2013	1	4	3	2	1	2	3	1143
3450	BPs38	2014	1	2	14	2	3	1	3	1144
3451	BPs39	2014	1	2	6	2	1	2	3	1145
3452	BPs40	2014	1	12	6	2	20	1	3	1146
3453	BPs41	2014	1	12	6	2	1	8	3	1147
3454	BPs14	2011	1	2	6	2	1	2	57	1136
4422	BPs61	2014	1	12	6	2	1	1	3	1132
4423	BPs66	2015	1	2	6	4	1	2	57	1135
4424	BPs70	2015	1	2	6	2	1	8	3	1137
4425	BPs71	2015	1	2	14	2	5	2	1	1434
4426	BPs72	2015	1	4	6	2	1	1	1	1435
4427	BPs76	2015	1	2	6	2	1	2	57	1136
4428	BPs83	2015	1	2	6	2	1	8	3	1137
4429	BPs80	2015	1	4	3	2	1	2	3	1143
4430	BPs89	2015	1	2	14	2	5	2	3	1436
4431	BPs96	2015	1	2	6	2	1	8	3	1137
4432	BPs98	2015	4	4	3	2	1	1	3	1437
4433	BPs85	2015	1	2	6	2	1	8	3	1137
4434	BPs58	2014	1	2	14	2	5	2	1	1434
4435	BPs60	2014	1	12	6	2	1	1	3	1132
4436	BPs63	2014	1	12	6	2	1	8	3	1147
4437	BPs65	2015	1	2	6	2	1	8	3	1137
4438	BPs67	2015	4	2	6	2	1	1	3	1140
4439	BPs69	2015	1	1	6	2	1	1	1	912
4440	BPs74	2015	1	2	14	2	5	2	1	1434
4441	BPs77	2015	1	12	6	2	1	8	3	1147
4442	BPs78	2015	1	2	124	2	1	8	3	1438
4443	BPs81	2015	1	2	6	2	1	2	57	1136
4444	BPs84	2015	18	4	3	4	1	1	1	1439
4445	BPs86	2015	1	2	6	2	1	8	3	1137
4446	BPs88	2015	1	2	6	2	1	1	3	655
4447	BPs91	2015	1	12	6	2	1	1	3	1132
4448	BPs94	2015	1	2	6	4	1	2	57	1135
4449	BPs97	2015	1	2	6	2	1	8	3	1137
4450	BPs99	2015	1	2	6	2	1	8	3	1137
4451	BPs101	2015	1	4	3	2	1	2	1	594
4452	BPs100	2015	1	2	6	2	1	8	3	1137
4453	BPs102	2015	1	16	13	4	6	21	1	132

MLST = multilocus sequence typing; ST = sequence types. Available for download from https://pubmlst.org/bpseudomallei/.
